# Vessel-derived angiocrine IGF1 promotes Meckel's cartilage proliferation to drive jaw growth during embryogenesis

**DOI:** 10.1242/dev.190488

**Published:** 2020-06-11

**Authors:** Ceilidh Marchant, Peter Anderson, Quenten Schwarz, Sophie Wiszniak

**Affiliations:** 1 Centre for Cancer Biology, University of South Australia and SA Pathology, Adelaide, SA 5000, Australia; 2 Australian Craniofacial Unit, Women's and Children's Hospital, North Adelaide, SA 5006, Australia

**Keywords:** IGF, Angiocrine, Craniofacial, Cartilage, Blood vessel, Mouse

## Abstract

Craniofacial development is a complex morphogenic process that requires highly orchestrated interactions between multiple cell types. Blood vessel-derived angiocrine factors are known to promote proliferation of chondrocytes in Meckel's cartilage to drive jaw outgrowth, however the specific factors controlling this process remain unknown. Here, we use *in vitro* and *ex vivo* cell and tissue culture, as well as genetic mouse models, to identify IGF1 as a novel angiocrine factor directing Meckel's cartilage growth during embryonic development. We show that IGF1 is secreted by blood vessels and that deficient IGF1 signalling underlies mandibular hypoplasia in *Wnt1-Cre*; *Vegfa^fl/fl^* mice that exhibit vascular and associated jaw defects. Furthermore, conditional removal of IGF1 from blood vessels causes craniofacial defects including a shortened mandible, and reduced proliferation of Meckel's cartilage chondrocytes. This demonstrates a crucial angiocrine role for IGF1 during craniofacial cartilage growth, and identifies IGF1 as a putative therapeutic for jaw and/or cartilage growth disorders.

## Abstract (precis)

**Highlighted Article:** This study shows IGF1 is secreted directly from endothelial cells in an angiocrine manner to instruct proliferation of Meckel's cartilage, underpinning correct jaw growth and extension during development.

## INTRODUCTION

Insufficient growth of the lower jaw (mandibular hypoplasia or micrognathia) is a common congenital malformation occurring in isolation or as part of the complex phenotypes underpinning craniofacial syndromes. Extension of the jaw during embryonic development is controlled by proliferation of chondrocytes within Meckel's cartilage, which acts as a scaffold for mandibular bone formation (Ramaesh and Bard, 2003). A combination of cell intrinsic and paracrine signals regulate chondrocyte proliferation, with blood vasculature recently found as a novel source of mitogenic signals (Wiszniak et al., 2015). Indeed, vascular defects also correlate with mandibular hypoplasia in patients with hemifacial microsomia, in which mandible defects are a prominent feature (Wiszniak et al., 2015).

As well as providing oxygen and nutrients to tissues, blood vessels have the capacity to secrete angiocrine factors directly from the endothelial or mural cell components of the vessel walls to instruct tissue morphogenesis (Rafii et al., 2016). Angiocrine factors influence a range of processes such as regeneration, organogenesis, stem-cell maintenance, cell differentiation and tumour growth (Cao et al., 2017; Hu et al., 2014; Lammert et al., 2001; Pedrosa et al., 2015; Rafii et al., 2016; Ramasamy et al., 2014). Which angiocrine factor(s) regulates chondrocyte proliferation in Meckel's cartilage is currently unknown.

Here, we show that IGF1 is an angiocrine factor secreted from blood vessels that can promote chondrocyte proliferation *in vitro* and *in vivo*. The IGF1 signalling pathway is downregulated in Meckel's cartilage of a mouse model with mandibular blood vessel defects and associated mandibular hypoplasia. We further show that genetic removal of IGF1 from endothelial cells reduces proliferation of Meckel's cartilage chondrocytes and compromises jaw outgrowth during development, providing unequivocal proof of an angiocrine requirement for IGF1 during jaw development. Finally, addition of recombinant IGF1 protein is able to recover the proliferation defects observed in Meckel's cartilage of *Wnt1-Cre;Vegfa^fl/fl^* mice. Together, this provides the first evidence of a role for angiocrine IGF1 during embryonic development *in vivo*, and provides insight into the complex mechanisms of mandibular growth.

## RESULTS AND DISCUSSION

### IGF1 is an angiocrine factor secreted from blood vessels that promotes Meckel's chondrocyte proliferation *in vitro*

**Fig. 1.**
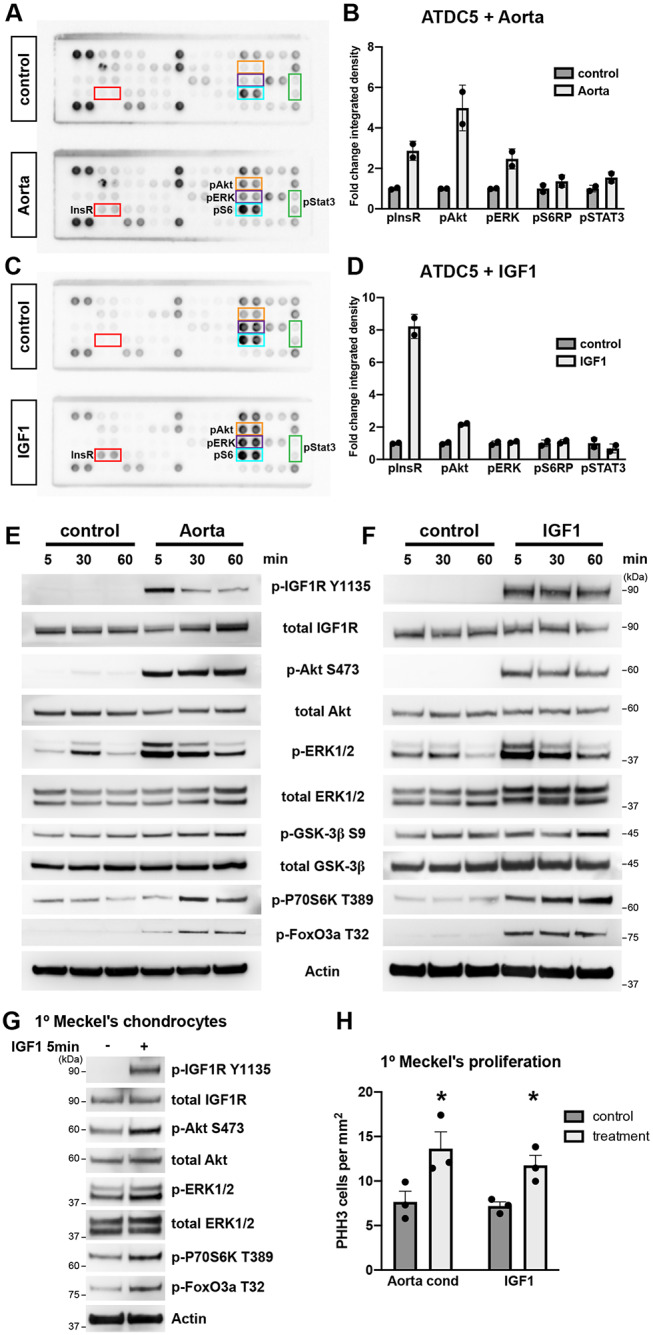
**Aorta-conditioned media activates the IGF signalling pathway in chondrocytes.** (A) Phospho-receptor tyrosine kinase array containing duplicate spots of 39 receptor tyrosine kinases and downstream signalling nodes. ATDC5 cells were stimulated with control- or aorta-conditioned media for 5 min, and cell lysates subjected to phospho-array. Coloured boxes highlight array spots that showed differences in phosphorylation between control and aorta-conditioned media treatment. (B) Quantitation of selected array spots in A. Data are mean±s.d. of integrated density measurement between duplicate spots. (C) Phospho-receptor tyrosine kinase array of ATDC5 cells stimulated with recombinant IGF1 (1000 ng/ml) for 5 min. (D) Quantitation of selected array spots in C. (E) Western blot of ATDC5 cells stimulated with control- or aorta-conditioned media for the indicated time periods. (F) Western blot of ATDC5 cells stimulated with recombinant IGF1 (1000 ng/ml) for the indicated time periods. (G) Western blot of primary Meckel's cartilage chondrocytes stimulated with recombinant IGF1 (1000 ng/ml) for 5 min. (H) Proliferation of primary Meckel's cartilage chondrocytes upon stimulation with aorta-conditioned media or recombinant IGF1 (1000 ng/ml) for 5 days. Graph represents mean number of PHH3-positive cells detected per mm^2^ of cells grown as a monolayer. Data are mean+s.e.m. from *n*=3 independent cell isolations/experiments. *P<0.05 (paired two-tailed *t*-test).

Secreted angiocrine factors from blood vessels are essential for promoting proliferation of Meckel's cartilage chondrocytes during a crucial time period of embryonic development (Wiszniak et al., 2015). We have previously shown that conditioned media from aortic rings contains factors that promote chondrocyte proliferation (Wiszniak et al., 2015), however the identity of these angiocrine factor(s) remains unknown. To identify these factor(s), the widely used murine chondrogenic model cell line ATDC5 (Shukunami et al., 1996; Yao and Wang, 2013) was stimulated with aorta-conditioned media for 5 min, and ATDC5 cell lysates were subjected to a receptor tyrosine kinase signalling antibody array to analyse receptor activation and downstream signalling pathways. Notably, treatment with aorta-conditioned media led to activation of the insulin receptor (IR), as well as Akt, ERK1/2 and Stat3 (Fig. 1A,B). This suggests that ligands for the IR are present in aorta-conditioned media. The IR has multiple ligands, including: insulin 1, insulin 2, IGF1 and IGF2 (Nakae et al., 2001). Quantitative RT-PCR revealed high expression of *Igf1*, minimal expression of *Igf2*, and no detectable expression of *Ins1* or *Ins2* in aortic ring tissue (Fig. S1), suggesting that IGF1 is the likely angiocrine ligand secreted from the aortic rings. ATDC5 cells were stimulated with recombinant IGF1, which showed a similar profile of IR and downstream signalling pathway activation by phospho-array (Fig. 1C,D), further suggesting IGF1 as a likely angiocrine factor present in aorta-conditioned media.

Insulin-like growth factor (IGF) signalling is essential for proper growth of the cartilage skeleton, as evidenced by the dramatic skeletal phenotypes observed in *Igf1R^−/−^*, *Igf1^−/−^*, *Igf2^−/−^* (full knockout) (Baker et al., 1993; Liu et al., 1993) and *Col2a1-Cre*; *Igf1R^fl/fl^* (chondrocyte-specific IGF1R knockout) (Wang et al., 2011) mouse embryos. However, removal of IGF1 specifically in chondrocytes (*Col2a1-Cre*; *Igf1^fl/fl^*) causes only mild skeletal defects in adult mice (Govoni et al., 2007), suggesting an endocrine/paracrine requirement for IGF1 in controlling chondrocyte growth during development. The precise source of IGF1, important for controlling Meckel's cartilage growth, has remained unclear, suggesting that IGF1 may be required in an angiocrine manner.

To investigate the IGF1 signalling pathway in detail, ATDC5 cells were stimulated with aorta-conditioned media over a time course from 5 to 60 min, and active signalling assessed by western blot. Robust phosphorylation of the IGF1R was observed upon aorta-conditioned media treatment, as well as downstream signalling components including Akt, ERK1/2 (Mapk3/1), Gsk3β, p70S6K (Rps6kb1) and FoxO3a (Foxo3) (Fig. 1E). This closely mirrored the IGF1R signalling components activated in ATDC5 cells upon stimulation with recombinant IGF1 (Fig. 1F), although there were minor differences in the timing kinetics and level of phosphorylation of some substrates between the two conditions, such as in IGF1R, p70S6K and FoxO3a. This may represent differences in the concentration of IGF1 present in the media of both treatments, as well as the possible presence of interacting factors in aorta-conditioned media, such as IGF-binding proteins (IGFBPs) which may modulate IGF1 signalling activity. As well as the ATDC5 cell line, primary Meckel's cartilage chondrocytes isolated from embryonic tissue showed a similar pattern of substrate activation when stimulated with recombinant IGF1 (Fig. 1G).

Aorta-conditioned media was previously shown to promote proliferation of primary Meckel's cartilage chondrocytes grown *in vitro* (Wiszniak et al., 2015 and Fig. 1H) and, importantly, recombinant IGF1 was able to induce chondrocyte proliferation to a similar extent (Fig. 1H). Together, this data supports a role for angiocrine IGF1 present in aorta-conditioned media to promote Meckel's cartilage proliferation.

### IGF signalling is downregulated in Meckel's cartilage of mice with mandibular hypoplasia

**Fig. 2.**
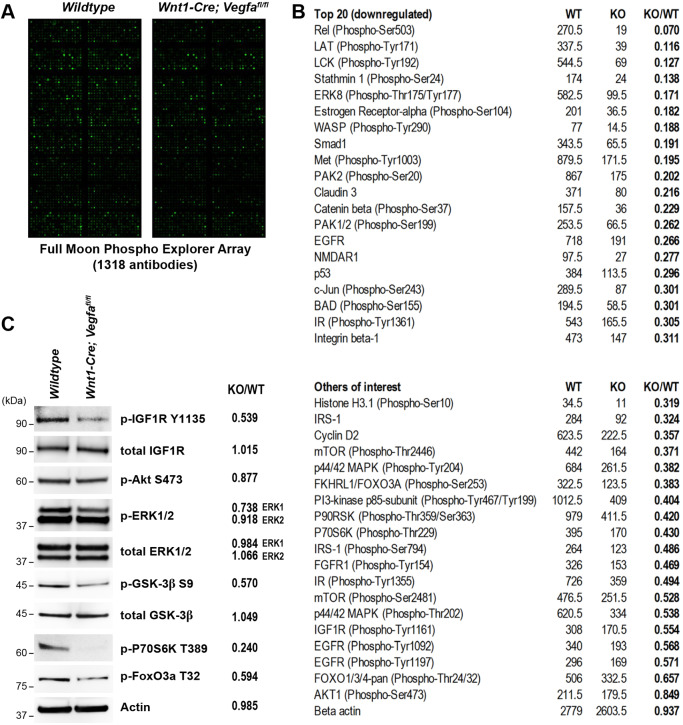
**IGF signalling is downregulated in Meckel's cartilage of *Wnt1-Cre; Vegfa^fl/fl^* mice.** (A) Phospho Explorer antibody array of dissected Meckel's cartilage tissue from E13.5 wild-type and *Wnt1-Cre; Vegfa^fl/fl^* embryos. (B) Selection of targets identified by the antibody array screen. Values represent mean pixel intensity calculated for each array spot using GenePix software for wild-type (WT) and *Wnt1-Cre; Vegfa^fl/fl^* (KO) arrays. Fold change (KO/WT) is calculated in the far-right column. Top panel lists the top 20 most downregulated antibody substrates in KO versus wild-type tissue. Bottom panel highlights other targets of interest from the antibody array list. (C) Western blot of dissected Meckel's cartilage tissue from E13.5 wild-type and *Wnt1-Cre; Vegfa^fl/fl^* embryos. Panel on the right represents fold change (KO/WT) of western blot quantitation by integrated density measurement of bands.

To investigate whether angiocrine IGF1 plays a role in Meckel's cartilage development *in vivo*, Meckel's cartilage from E13.5 wild-type and *Wnt1-Cre*; *Vegfa^fl/fl^* embryos (which exhibit mandibular hypoplasia due to loss of mandibular artery-derived angiocrine factors; Wiszniak et al., 2015) were microdissected and protein lysates analysed by Full Moon Phospho Explorer Array containing 1318 signalling-related antibodies (Fig. 2A). Notably, the IR was one of the top differentially phosphorylated receptors, with ∼70% reduction in phosphorylation in *Wnt1-Cre*; *Vegfa^fl/fl^* embryos (Fig. 2B). The IGF1R showed a ∼50% reduction in phosphorylation in *Wnt1-Cre*; *Vegfa^fl/fl^* Meckel's cartilage, as well as many downstream targets of the IGF1 signalling pathway, such as p44/42 MAPK (ERK1/2), mTOR, FOXO3a, PI3K, p70S6K and IRS1 (Fig. 2B). To validate the array data, dissected Meckel's cartilage tissue from E13.5 wild-type and *Wnt1-Cre*; *Vegfa^fl/fl^* embryos was analysed by western blot to investigate the IGF1 signalling pathway and downstream effectors. This revealed a reduction in IGF1R, ERK1, Gsk3β, p70S6K and FoxO3a phosphorylation in Meckel's cartilage *in vivo* (Fig. 2C). In contrast to stimulation of chondrocytes with recombinant IGF1 (Fig. 1), there was no change in steady-state levels of Akt or ERK2 phosphorylation in *Wnt1-Cre*; *Vegfa^fl/fl^* Meckel's cartilage (Fig. 2B,C). Taken together, this unbiased array analysis followed by validation using targeted IGF1 signalling pathway antibodies provides strong evidence for disruption of IGF1 signalling in *Wnt1-Cre*; *Vegfa^fl/fl^* Meckel's cartilage *in vivo*. Given the lack of mandibular vasculature in *Wnt1-Cre*; *Vegfa^fl/fl^* embryos, this suggests blood vessels may be the angiocrine source of IGF1 driving jaw growth.

As well as reduced activation of the IR and IGF1R in *Wnt1-Cre*; *Vegfa^fl/fl^* Meckel's cartilage, several other receptors and signalling nodes showed reduced phosphorylation (Fig. 2B). The most severely affected substrates (Rel, LAT, LCK) have well-known roles in lymphocyte function and maturation (Kontgen et al., 1995; Zhang et al., 1999; Molina et al., 1992), but their role in chondrocyte development remains unclear. Reductions of these substrates may be a consequence of decreased vasculature and hence fewer blood cells present in *Wnt1-Cre*; *Vegfa^fl/fl^* tissue samples compared with wild-type samples. Other receptor tyrosine kinases exhibiting reduced phosphorylation in *Wnt1-Cre*; *Vegfa^fl/fl^* Meckel's cartilage include FGFR1 and EGFR (Fig. 2B). Neural crest-specific knockout of FGFR1 causes craniofacial defects including cleft palate and micrognathia (Wang et al., 2013), suggesting FGFR1 signalling may also contribute to mandible extension. EGFR knockout mice exhibit profound micrognathia and severe dysmorphogenesis of Meckel's cartilage development (Miettinen et al., 1999). This suggests ligands for FGFR1 and EGFR, such as FGFs, EGF and TGF-α, may serve as additional angiocrine factors important for craniofacial development, and may warrant future investigation.

### IGF1 is required from blood vessels for jaw extension

**Fig. 3.**
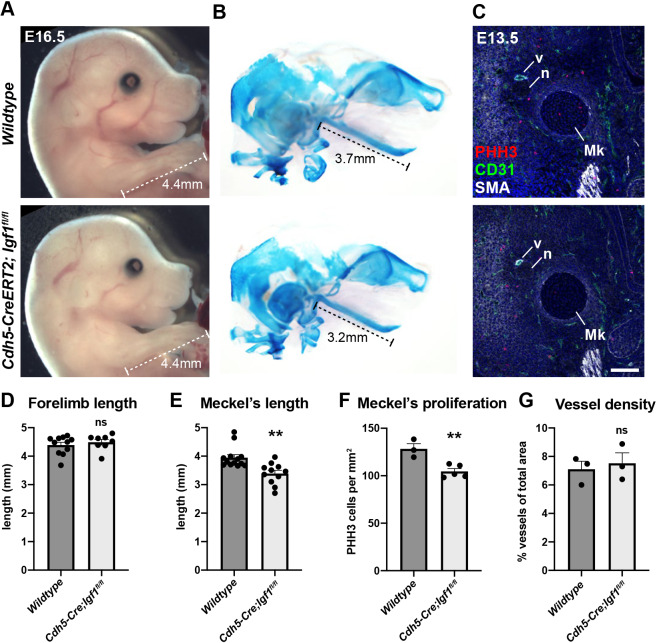
**Meckel's cartilage growth is reduced in *Cdh5-CreERT2; Igf1^fl/fl^* mice.** (A) E16.5 wild-type and *Cdh5-CreERT2; Igf1^fl/fl^* embryos. Conditional knockout of *Igf1* was induced with tamoxifen at E11.5, E12.5 and E13.5. Dashed line indicates measurement used to calculate forelimb length. (B) Alcian Blue skeletal staining of heads from E16.5 wild-type and *Cdh5-CreERT2; Igf1^fl/fl^* embryos. Dashed line indicates measurement used to calculate Meckel's cartilage length. (C) Frontal section through the mandible of E13.5 wild-type and *Cdh5-CreERT2; Igf1^fl/fl^* embryos immunostained for PHH3, CD31 and smooth muscle actin (SMA). Mk, Meckel's cartilage; n, nerve; v, mandibular vessel. Scale bar: 100 µm. (D) Mean forelimb length of E16.5 wild-type and *Cdh5-CreERT2; Igf1^fl/fl^* embryos, calculated from measurement shown in A. *n*=11 wild-type and *n*=8 *Cdh5-CreERT2; Igf1^fl/fl^* embryos. (E) Mean Meckel's cartilage length of E16.5 wild-type and *Cdh5-CreERT2; Igf1^fl/fl^* embryos, calculated from measurement shown in B. *n*=13 wild-type and *n*=11 *Cdh5-CreERT2; Igf1^fl/fl^* embryos. **P=0.0014 (unpaired two-tailed *t*-test). (F) Mean Meckel's cartilage proliferation in E13.5 wild-type and *Cdh5-CreERT2; Igf1^fl/fl^* embryos, expressed as number of PHH3-positive cells per mm^2^ of Meckel's cartilage tissue area from sections shown in C. *n*=3 wild-type and *n*=5 *Cdh5-CreERT2; Igf1^fl/fl^* embryos. **P=0.0056 (unpaired two-tailed *t*-test). (G) Mean vessel density in E13.5 wild-type and *Cdh5-CreERT2; Igf1^fl/fl^* embryos, expressed as the percentage of total tissue area positive for CD31 immunostaining from sections shown in C. *n*=3 wild-type and *n*=3 *Cdh5-CreERT2; Igf1^fl/fl^* embryos. Data are mean+s.e.m. ns, not significant (unpaired two-tailed *t*-test).

To definitively test the angiocrine requirement for IGF1 in jaw growth, *Igf1* was conditionally removed from endothelial cells using the *Cdh5-CreERT2* driver, with knockout induced by tamoxifen injection at E11.5, E12.5 and E13.5, which is just before the developmental expansion of Meckel's cartilage (Wiszniak et al., 2015). Knockout of *Igf1* from the mandibular vessel and surrounding microvasculature was confirmed by assessing *Igf1* mRNA expression by *in situ* hybridisation in wild-type versus *Cdh5-CreERT2*; *Igf1^fl/fl^* embryos (Fig. S2) and by crossing *Cdh5-CreERT2* mice to *R26R lacZ* reporter mice, which confirmed genetic recombination in the mandibular vessel (Fig. S3). Although the body size of E16.5 *Cdh5-CreERT2*; *Igf1^fl/fl^* embryos appeared unaffected compared with wild-type controls (quantified by forelimb length in Fig. 3A,D), the head was disproportionately smaller (Fig. 3A) and Meckel's cartilage was significantly reduced in length, as assessed by whole-mount Alcian Blue staining of skulls (Fig. 3B,E). Underpinning the reduction in length of Meckel's cartilage, proliferation in Meckel's cartilage was reduced at E13.5 in *Cdh5-CreERT2*; *Igf1^fl/fl^* embryos (Fig. 3C,F). This was in the absence of gross blood vessel defects as assessed by quantifying CD31^+^ blood vessel density at E13.5 (Fig. 3G).

Taken together, this suggests that endothelial cells provide an essential source of IGF1 that is secreted in an angiocrine manner to instruct proliferation of Meckel's cartilage chondrocytes. Given that Meckel's cartilage directly serves as the scaffold for mandible bone ossification, this proliferation underpins correct jaw lengthening and extension during embryonic development. Why angiocrine IGF1 does not affect other cartilage growth centres throughout the embryo, such as the forelimb, remains to be investigated but may reflect differences in chondrocytes of neural crest versus mesenchymal origin. Previous studies have shown IGF1 to act as an angiocrine factor in an *in vitro* assay to induce muscle progenitor proliferation (Christov et al., 2007), and in a cancer model to promote chemoresistance and expansion of tumour stem-like cells (Cao et al., 2017); however, our study is the first to demonstrate a role for angiocrine IGF1 during embryonic development *in vivo*.

Interestingly, the jaw outgrowth defect in *Cdh5-CreERT2; Igf1^fl/fl^* mice only partially phenocopied that previously identified in *Wnt1-Cre; Vegfa^fl/fl^* mice (Wiszniak et al., 2015). One possibility is that the presumed removal of multiple angiocrine factors as a result of mandibular vessel absence in *Wnt1-Cre; Vegfa^fl/fl^* mice has a more pronounced combinatorial effect on Meckel's cartilage growth than the removal of a single angiocrine factor (IGF1). Given that the embryonic growth of *Igf1^−/−^;Igf2^−/−^* double knockout embryos is further reduced compared with single knockouts alone (Baker et al., 1993; Liu et al., 1993), this suggests IGF2 may be able to partially compensate for loss of angiocrine IGF1. Indeed, future studies may investigate IGF2 as an additional angiocrine factor involved in Meckel's cartilage growth. As well as by the vasculature, *Igf1* is expressed by the investing mesenchyme in close proximity to Meckel's cartilage (Fig. S2), which remains highly expressed in *Cdh5-CreERT2; Igf1^fl/fl^* embryos. Why this expression does not compensate for lack of IGF1 in endothelial cells remains to be investigated, but may involve the activity and expression of IGFBPs which can enhance or attenuate the signalling activity of IGFs (Allard and Duan, 2018), and remain to be studied in the context of Meckel's cartilage development.

### Addition of IGF1 recovers Meckel's cartilage proliferation in growth-deficient mandibles

**Fig. 4.**
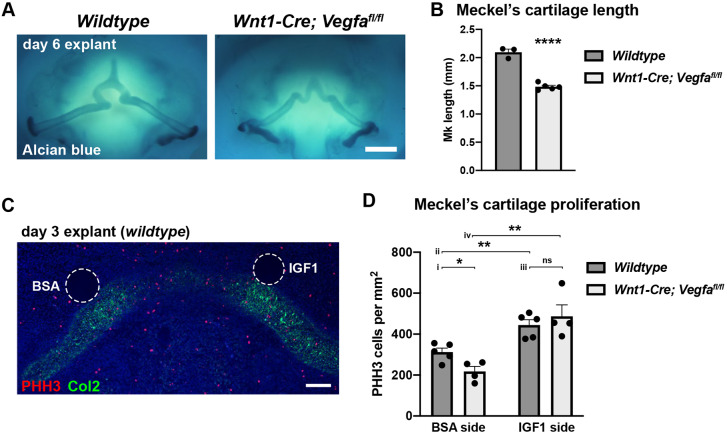
**IGF1 addition recovers Meckel's cartilage proliferation in *Wnt1-Cre; Vegfa^fl/fl^* mandible explants.** (A) Mandible explants from wild-type and *Wnt1-Cre; Vegfa^fl/fl^* embryos cultured for 6 days and stained with Alcian Blue. Scale bar: 0.5 mm. (B) Mean Meckel's cartilage length quantified from images shown in A. Length was measured per side from *n*=3 wild-type and *n*=5 *Wnt1-Cre; Vegfa^fl/fl^* mandible explants. Data are mean+s.e.m., ****P<0.0001 (unpaired two-tailed *t*-test). (C) Section through a wild-type mandible explant after 3 days of culture demonstrating positioning of BSA (left) and IGF1 (right) beads that were implanted on day 0 of culture. Section has been immunostained for PHH3 and collagen type 2 (Col2) to indicate Meckel's cartilage. Scale bar: 100 µm. (D) Mean Meckel's cartilage proliferation in wild-type and *Wnt1-Cre; Vegfa^fl/fl^* explants quantified from images as shown in C. Proliferation was calculated as the number of PHH3^+^ cells per mm^2^ area of Col2^+^ cartilage tissue directly adjacent the implanted bead (within 200 µm radius). Data are mean+s.e.m. from *n*=5 wild-type and *n*=4 *Wnt1-Cre; Vegfa^fl/fl^* explants. *P=0.018 (i); **P=0.0039 (ii) and 0.0046 (iv); ns, not significant (iii) (unpaired two-tailed *t*-test).

*Wnt1-Cre; Vegfa^fl/fl^* embryos exhibit reduced Meckel's cartilage growth owing to a lack of blood vessels and associated angiocrine factors (Wiszniak et al., 2015). This phenotype remained consistent when mandible explants from E11.5 *Wnt1-Cre; Vegfa^fl/fl^* embryos were grown *ex vivo*, with Meckel's cartilage length significantly reduced compared with wild-type explants after 6 days of culture (Fig. 4A,B). This growth deficiency was underpinned by a reduction in proliferation in Meckel's cartilage chondrocytes as assessed at 3 days of culture (Fig. 4D, i). To test the ability of exogenous IGF1 to recover proliferation in growth-deficient mandibles, beads soaked in either bovine serum albumin (BSA) (control) or recombinant IGF1 were implanted directly adjacent to the developing Meckel's cartilage at the time of explant (Fig. 4C). Sections through the mandibles after 3 days of culture revealed an increase in proliferation in Meckel's cartilage on the IGF1 treated side in wild-type mandibles (Fig. 4D, ii), as well as in *Wnt1-Cre; Vegfa^fl/fl^* mandibles, in which proliferation was recovered to the same extent as in the wild type (Fig. 4D, iii and iv). This further supports the hypothesis that lack of angiocrine IGF1 is responsible for the shortened mandibles observed in *Wnt1-Cre; Vegfa^fl/fl^* and *Cdh5-CreERT2; Igf1^fl/fl^* mice, and that paracrine factors, as well as cell-intrinsic mechanisms, play important instructive roles in Meckel's cartilage growth during development.

Mutations and deletions in *IGF1* (Woods et al., 1996) and *IGF1R* (Abuzzahab et al., 2003; Juanes et al., 2015; Okubo et al., 2003) can cause microcephaly and micrognathia in humans, and *IGF1R* duplication in one patient caused macroglossia and a large prominent chin (Okubo et al., 2003). This fits with an essential role for IGF1/IGF1R signalling in craniofacial and jaw development, and is consistent with IGF1 being required in an angiocrine manner to mediate Meckel's cartilage growth. This presents the possibility of recombinant IGF1 administration, or manipulation of the IGF1R signalling pathway, being explored for potential therapeutic uses for individuals with jaw growth disorders.

### Conclusion

Here, we present the first evidence for IGF1 being required in an angiocrine manner during embryonic development *in vivo*. Our findings provide new insight into the molecular mechanisms underpinning jaw morphogenesis, and also propose characterisation of a role for angiocrine IGF1, and other novel angiocrine factors, more broadly in other developmental biological systems. The ability of IGF1 to recover proliferation in growth-deficient mandibles also provides proof-of-concept evidence that IGF1 may be a novel therapeutic for jaw and/or cartilage growth disorders.

## MATERIALS AND METHODS

### Animals

To obtain embryos of defined gestational ages, mice were mated in the evening, and the morning of vaginal plug formation was counted as embryonic day (E) 0.5. Pregnant dams were humanely euthanised at relevant days post vaginal plug detection by CO_2_ inhalation and cervical dislocation. To remove VEGF specifically in neural crest cells, *Wnt1-Cre; Vegfa^fl/+^* males were crossed to *Vegfa^fl/fl^* females (Danielian et al., 1998; Gerber et al., 1999). To remove IGF1 specifically in endothelial cells, *Cdh5-CreERT2;Igf1^fl/fl^* males were crossed to *Igf1^fl/fl^* females (Sorensen et al., 2009; Temmerman et al., 2010), and conditional knockout induced by intraperitoneal injection of 125 µl of 20 mg/ml tamoxifen dissolved in sunflower oil. To lineage trace *Cdh5-CreERT2*-positive cells, *Cdh5-CreERT2* mice were crossed to *R26R lacZ* reporter mice. In all experiments, tamoxifen injection was performed once daily at E11.5, E12.5 and E13.5. All experiments were carried out under the approval of the SA Pathology and University of South Australia Animal Ethics Committee (approved project number: 27-16).

### Cell culture

The murine pre-chondrogenic cell line ATDC5 (Shukunami et al., 1996) was a gift from Prof. Stan Gronthos (University of Adelaide, Australia), and has been verified for chondrogenic differentiation potential. Cells were cultured in DMEM/F12 with addition of 5% foetal calf serum (FCS). ATDC5 cells were grown to confluence and aortic ring-conditioned media, or recombinant human IGF1 (1 µg/ml), applied for the indicated time periods.

### Aortic ring-conditioned media

Aortic rings were cultured as previously described (Baker et al., 2011). Aortic rings were embedded in collagen and media (DMEM/F12 with 5% FCS) was applied on top of the collagen gel for 2 days, then removed and applied directly to ATDC5 or primary Meckel's cartilage cells.

### Primary Meckel's cartilage culture

Primary Meckel's cartilage chondrocytes were isolated using a protocol modified from Ishizeki et al. (1996). Briefly, Meckel's cartilage bars were dissected from E14.5 wild-type mouse embryos. Cartilage was digested with 0.15% trypsin (Gibco), 0.1% EDTA in DMEM for 30 min, washed with PBS, and then further digested with 0.15% collagenase type II (Worthington) in DMEM for 30 min, both at 37°C. Dissociated cells were plated onto 50 µg/ml fibronectin-coated 48-well culture plates at a density of 1×10^5^ cells per well. Cells were cultured in DMEM/F12 containing 5% FCS. For IGF1 or aorta-conditioned media stimulation experiments, cells were grown for 2 days of initial culture before treatment was applied. For proliferation analysis, treatment was applied for 5 days, with replenishment of growth media every second day.

### RTK array

ATDC5 cells were cultured and stimulated with aorta-conditioned media or recombinant human IGF1 for 5 min, then protein lysates prepared using Cell Lysis Buffer (Cell Signaling Technology #9803). The Pathscan RTK Signaling Antibody Array (Cell Signaling Technology #7982) was performed following the manufacturer's instructions. The array slide was analysed via chemiluminescent read-out using LAS4000 equipment (Fujifilm Life Sciences). Integrated density of array spots was quantified using ImageJ.

### Quantitative RT-PCR

Total RNA was isolated from tissue using Trizol (Ambion) and single-stranded cDNA was synthesised using the QuantiTect Reverse transcription kit (Qiagen). qPCR was performed with SYBR Green reagent (Qiagen) using the Rotor-Gene Q real-time PCR system (Qiagen). Primers used were as follows: *Igf1* F: TGGATGCTCTTCAGTTCGTG, *Igf1* R: CACTCATCCACAATGCCTGT, *Igf2* F: CGCTTCAGTTTGTCTGTTCG, *Igf2* R: GGGGTGGCACAGTATGTCTC, *Ins1* F: TATAAAGCTGGTGGGCATCC, *Ins1* R: CCAGCAGGGGTAGGAAGTG, *Ins2* F: TGGAGGCTCTCTACCTGGTG, *Ins2* R: GGTCTGAAGGTCACCTGCTC, *GAPDH* F: ACCCAGAAGACTGTGGATGG, *GAPDH* R: CAGTGAGCTTCCCGTTCA. Relative mRNA levels were quantified using the delta-delta CT method (normalised to *GAPDH*, and expressed relative to *IGF1* levels in aortic ring tissue). Each PCR was performed in technical triplicates, with three biological replicates. Error bars represent s.e.m. between biological replicates.

### Western blotting

Cells or tissue were lysed in NP-40 buffer [137 mM NaCl, 10 mM Tris (pH 7.4), 10% glycerol, 1% NP-40, 2 mM sodium fluoride and 2 mM sodium vanadate] containing Complete Protease Inhibitors (Roche). Meckel's cartilage tissue was sonicated to completely dissociate the tissue. Protein samples were separated by SDS-PAGE and transferred to Amersham Hybond-P PVDF membrane (GE Healthcare). Membranes were blocked in 5% skim milk powder in TBS with 0.1% Tween 20. The following primary antibodies were used: anti-Phospho-IGF-I Receptor β (Tyr1135) (Cell Signaling Technology #3918; RRID:AB_10548764) 1:500; anti-IGF-I Receptor β (Cell Signaling Technology #9750; RRID:AB_10950969) 1:500; anti-Phospho-Akt (Ser473) (Cell Signaling Technology #4060; RRID:AB_2315049) 1:500; anti-Akt (Cell Signaling Technology #9272; RRID:AB_329827) 1:500; anti- Phospho-p44/42 MAPK (Erk1/2) (Thr202/Tyr204) (Cell Signaling Technology #4370; RRID:AB_2315112) 1:500; anti-p44/42 MAPK (Erk1/2) (Cell Signaling Technology #9102; RRID:AB_330744) 1:500; anti-Phospho-GSK-3β (Ser9) (Cell Signaling Technology #5558; RRID:AB_10013750) 1:500; anti-GSK-3β (Cell Signaling Technology #9315; RRID:AB_490890) 1:500; anti-Phospho-p70 S6 Kinase (Thr389) (Cell Signaling Technology #9205; RRID:AB_330944) 1:500; anti-Phospho-FoxO1 (Thr24)/FoxO3a (Thr32)/FoxO4 (Thr28) (4G6) (Cell Signaling Technology #2599; RRID:AB_2106814) 1:500; anti-β-Actin (Sigma-Aldrich #A5441; RRID:AB_476744) 1:5000. Secondary antibodies used were: donkey anti-mouse-HRP (Jackson ImmunoResearch) 1:5000; donkey anti-rabbit-HRP (Jackson ImmunoResearch) 1:5000. Membranes were analysed using Amersham ECL Prime reagent (GE Healthcare) and LAS4000 detection equipment. Quantitation of western blot was performed by measuring integrated density of bands using ImageJ.

### Immunohistochemistry

Cells, embryos and tissue explants were fixed in 4% paraformaldehyde. Embryos and tissue explants were cryosectioned at 12 µm. Cryosections and fixed cells were blocked in 10% DAKO serum-free blocking solution in PBST (PBS + 0.1% Triton X-100). The following primary antibodies were used: anti-Phospho Histone H3 Ser10 (PHH3) (Millipore #06-570; RRID:AB_310177) 1:500; anti-CD31 (BioLegend #102502; RRID:AB_312909) 1:150; anti-αSmooth muscle actin (SMA) (Sigma-Aldrich #A2547; RRID:AB_476701) 1:2000; anti-Collagen type 2 (Col2) (Developmental Studies Hybridoma Bank #II-II6B3-s; RRID:AB_528165) 1:25. The following secondary antibodies were used: anti-rabbit AlexaFluor 488 (Life Technologies #A21206) 1:200; anti-rabbit AlexaFluor 555 (Life Technologies #A31572) 1:200; anti-mouse AlexaFluor 647 (Life Technologies #A31571) 1:200; anti-mouse AlexaFluor 488 (Life Technologies #A21202) 1:200; anti-rat AlexaFluor 488 (Life Technologies #A21208) 1:200. Tissue sections were mounted in Prolong Diamond Antifade with DAPI (Life Technologies #P36962). Images were acquired using a Zeiss LSM 800 confocal microscope.

### Analysis of proliferation

Fixed cells or cryosections were immunostained for PHH3 and imaged on Zeiss LSM 800. Zen software (Zeiss) was used to measure the area (mm^2^) of the region of interest, and the number of PHH3^+^ nuclei lying within the region of interest were counted. For primary Meckel's chondrocyte cell proliferation, nine regions of interest were randomly selected from control and treatment wells and quantified, for *n*=3 independent experiments. For cryosections, the outline of Meckel's cartilage was traced to create the region of interest. For E13.5 embryos, ∼40 sections were quantified per embryo, for a minimum of *n*=3 wild-type and *Cdh5-CreERT2; Igf1^fl/fl^* embryos. For mandible explants, approximately eight sections were quantified per treatment side for a minimum of *n*=4 independent explants. Col2^+^ chondrocytes within a 200 µm radius of the implanted bead were included in the quantified area.

### Analysis of vessel density

Cryosections were immunostained for CD31 and imaged on Zeiss LSM 800. A region of interest ∼0.14 mm^2^ was drawn directly adjacent Meckel's cartilage, encompassing the major mandibular artery and nearby capillaries using Zen software (Zeiss). The outline of all CD31^+^ vasculature within the region of interest was manually traced and internal area measured. Vessel density was expressed as the area of CD31^+^ staining as a percentage of total area of the region of interest. Four regions of interest were measured per embryo on four different cryosections, from *n*=3 embryos.

### Full Moon Array

Meckel's cartilage was dissected from E13.5 wild-type and *Wnt1-Cre;Vegfa^fl/fl^* embryos and snap frozen in Extraction Buffer (Full Moon Biosystems) with the addition of 2 mM sodium fluoride, 2 mM sodium vanadate and Complete Protease Inhibitors (Roche). Meckel's cartilage tissue from *n*=13 wild-type and *Wnt1-Cre; Vegfa^fl/fl^* embryos was pooled and prepared following the manufacturer's recommendations. Lysates were applied to Phospho Explorer Antibody Array (Full Moon Biosystems #PEX100) and analysed using GenePix4000B scanner and software (Molecular Devices). Data are expressed as mean intensity values for each spot, as well as fold change of *Wnt1-Cre; Vegfa^fl/fl^* (KO)/wild type.

### Skeletal staining

Skulls were fixed in 95% ethanol, and Alcian Blue staining of cartilage was performed as previously described (Wiszniak et al., 2013).

### Mandible explants

The mandibular arch was dissected from E11.5 embryos and placed oral-epithelium-side up onto MCE membrane filter 0.8 µm (Millipore #AAWP04700) supported by Millicell cell culture inserts (0.4 µm, 30 mm diameter; Millipore #PICM0RG50) floating atop BGJb culture medium (Gibco #12591038) with the addition of 100 µg/ml ascorbic acid in 6-well tissue culture plates. Culture media was carefully applied to cover the explants such that the tissue sat at the air-media interface. Agarose beads (Affi-Gel Blue Gel, Bio-Rad #153-7302) were washed in PBS, then all liquid removed and beads air-dried slightly, before addition of 10% BSA or recombinant human IGF1 100 µg/ml (R&D Systems #291-G1), and incubated for at least 30 min at room temperature. Beads were implanted into the mandible tissue at time of explant. Explants were cultured up to 6 days, with exchange of fresh culture medium every second day. For Alcian Blue staining, explants were fixed on the membrane in 95% ethanol, and incubated in staining solution (0.02% Alcian Blue, 5% glacial acetic acid in 70% ethanol) for 2 days, then cleared in 1% KOH for several hours.

### *In situ* hybridisation

Section *in situ* hybridisation was performed as previously described (Schwarz et al., 2004). Riboprobe was transcribed from a plasmid containing the cDNA sequence for *Igf1*.

### β-Galactosidase staining

Cryosections were incubated in staining solution (19 mM sodium dihydrogen phosphate, 81 mM disodium hydrogen phosphate, 2 mM MgCl_2_, 5 mM EGTA, 0.01% sodium deoxycholate, 0.02% NP-40, 5 mM potassium ferricyanide, 5 mM potassium ferrocyanide and 1 mg/ml X-gal substrate) at 37°C until blue staining was sufficient. Sections were counterstained with Eosin.

## Supplementary Material

Supplementary information

Reviewer comments
