# Supplementary material for: Vessel-derived angiocrine IGF1 promotes Meckel's cartilage proliferation to drive jaw growth during embryogenesis
Source: Development. 2020 Jun 11;147(11):dev190488. doi: 10.1242/dev.190488 (PMC7295590; doi:10.1242/dev.190488)
Supplement: Supplementary information [file develop-147-190488-s1.pdf]

Supp Figure 1

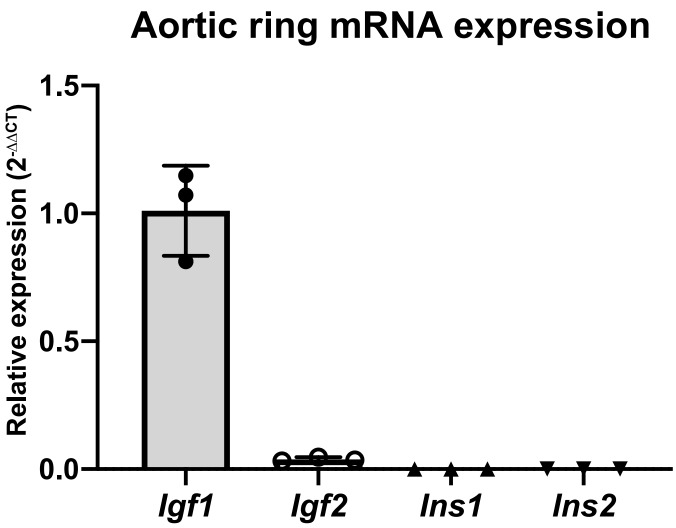

Figure S1:

mRNA expression of *Igf* and *Insulin* in aortic ring tissue.

Quantitative RT-PCR for *Igf1*, *Igf2*, *Ins1* and *Ins2* mRNA in aortic ring tissue. Graph represents relative concentrations of mRNAs calculated using the delta-delta CT method. mRNA levels for each gene were first normalised to *GAPDH* expression (ΔCT). Relative levels for each gene were then expressed relative to the level of *Igf1* (ΔΔCT). Fold change relative to *Igf1* was calculated with the equation $2^{-\Delta\Delta CT}$. Primer sets were independently validated in additional tissue samples (E14.5 whole embryo lysate) as a positive control.

Supp Figure 2

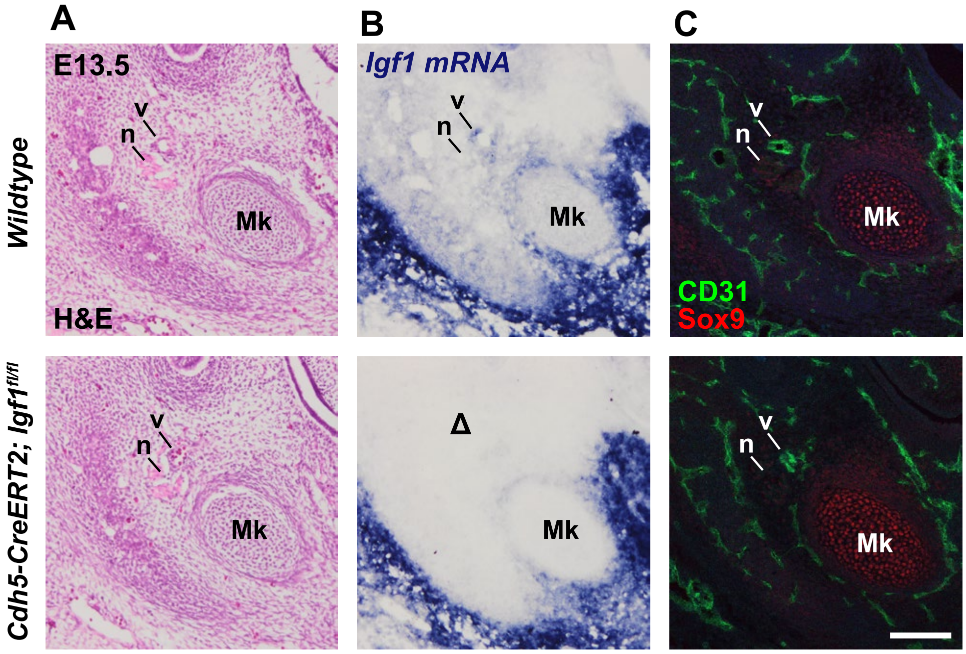

Figure S2:

Igf1 expression is reduced in *Cdh5-CreERT2; Igf1$^{fl/fl}$* embryos.

A: Frontal sections through the mandible of E13.5 wildtype and *Cdh5-CreERT2; Igf1$^{fl/fl}$* embryos stained for Haematoxylin and Eosin. Mk, Meckel's cartilage; n, nerve; v, mandibular vessel. **B:** Serial section with *in situ* hybridisation for *Igf1* mRNA. *Igf1* is reduced in expression around the mandibular vessel region (Δ) of *Cdh5-CreERT2;Igf1$^{fl/fl}$* embryos. **C:** Serial section immunostained for CD31 and Sox9. Scale bar = 100μm.

Supp Figure 3

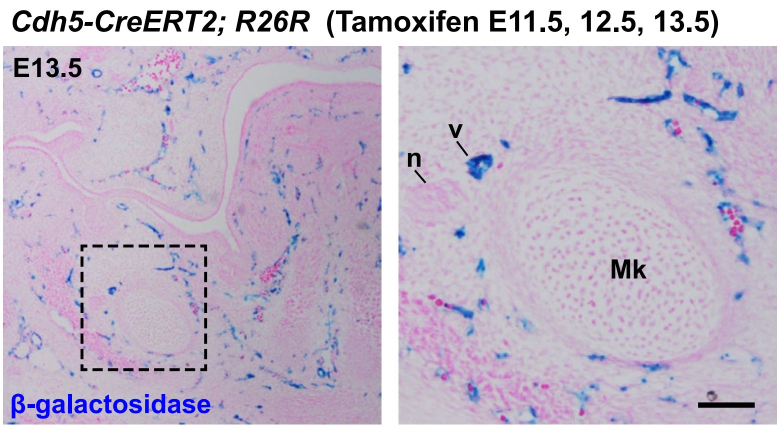

Figure S3:

Tamoxifen injection induces genetic recombination in the mandibular blood vasculature. Frontal section through the mandible of E13.5 *Cdh5-CreERT2; R26R* embryo, with tamoxifen injection at E11.5, E12.5 and E13.5 to induce *LacZ* reporter gene recombination. Tissue section has been stained with X-gal to show areas of β-galactosidase activity. β-galactosidase reporter gene expression is restricted to the vasculature, including the mandibular vessel (v). Mk, Meckel's cartilage; n, nerve. Scale bar = 50μm.
