# Supplementary material for: Vessel-derived angiocrine IGF1 promotes Meckel's cartilage proliferation to drive jaw growth during embryogenesis
Source: Development. 2020 Jun 11;147(11):dev190488. doi: 10.1242/dev.190488 (PMC7295590; doi:10.1242/dev.190488)
Supplement: Reviewer comments [file develop-147-190488_review_history.pdf]

Vessel-derived angiocrine IGF1 promotes Meckel's cartilage proliferation to drive jaw growth during embryogenesis

Ceilidh Marchant, Peter Anderson, Quenten Schwarz and Sophie Wiszniak
DOI: 10.1242/dev.190488

Editor: Patrick Tam

Review timeline

Original submission:	13 March 2020
Editorial decision:	14 April 2020
First revision received:	19 April 2020
Accepted:	23 April 2020

Original submission

First decision letter

MS ID#: DEVELOP/2020/190488

MS TITLE: Angiocrine IGF-1 promotes Meckel's cartilage proliferation to drive jaw growth during embryogenesis

AUTHORS: Ceilidh Marchant, Peter Anderson, Quenten Schwarz, and Sophie Wiszniak

I have now received all the referees reports on the above manuscript, and have reached a decision. The referees' comments are appended below, or you can access them online: please go to BenchPress and click on the 'Manuscripts with Decisions' queue in the Author Area.

The overall evaluation is positive and we would like to publish a revised manuscript in Development, provided that the referees' comments and the points noted the Editor (see Editor's Note appended below) can be satisfactorily addressed. Please attend to all of the reviewers' comments in your revised manuscript and detail them in your point-by-point response. If you do not agree with any of their criticisms or suggestions explain clearly why this is so.

We are aware that you may currently be unable to access the lab to undertake experimental revisions. If it would be helpful, we encourage you to contact us to discuss your revision in greater detail. Please send us a point-by-point response indicating where you are able to address concerns raised (either experimentally or by changes to the text) and where you will not be able to do so within the normal timeframe of a revision. We will then provide further guidance. Please also note that we are happy to extend revision timeframes as necessary.

Editor’s Note:

1. Provide a detailed description (in Results) or a revised legend to help the reader to navigate through the data presented in Figure 1A-D.
2. Inference of “closely mirrored the IGF-1R signalling component activate in ATDc5 cells with recombinant IGF-1””: Comment on the differences in the phosphorylation (timing and level) of p-IGF-IR Y1136, p-S6K T389 and p-Fox03a T32 between treatment of aorta-conditioned medium and recombinant IGF-1 (figure 1E-F)

© 2020. Published by The Company of Biologists under the terms of the Creative Commons Attribution License (https://creativecommons.org/licenses/by/4.0/).

3. Figure 2C and text: Clarify if ps6k-T389 (with reduced phosphorylation) is same as p70S6K (data not shown?), and if phosphorylation of ERK1/2 was significantly reduced. It would be informative to quantify the level of phosphorylation of the factors shown in Fig 2C.
4. Comment on the phenomenon of lack of effect of IGF-1 signals from the investing mesenchyme (Fig S2) on the growth of the Meckel's cartilage of the endothelial-IGF1 deficient Cdh5-CreERT2;Igf1-f/f embryo

Reviewer 1

Advance summary and potential significance to field

Angiocrine regulation of Igf1 might promote bone repair and regeneration.

Comments for the author

This is a well executed study demonstrating the angiocrine function of Igf1 in bone development worthy of publication.

Reviewer 2

Advance summary and potential significance to field

This is a well performed and rigorous study. It clearly demonstrates an interesting role for vessel-derived IGF-1 in promoting growth of Meckel's cartilage and hence the lower jaw. A number of sophisticated techniques are employed. Proteomic arrays identified Igfr-1 signaling and its downstream effectors in Meckel's cartilage, which are well validated from Westerns. Mouse genetics identify that Igfr-1 signaling depends on the presence of sufficient blood vessels, and conditional deletion of Igf-1 in the vasculature clearly reveals a role for vessel-derived Igf-1 in Meckel's growth.
Lastly, addition of Igf-1 to Meckel's explants rescues growth in vessel-deficient jaws. I found the paper to be an elegant example of how vessel-derived signals potentiate jaw growth and had only very minor comments.

Comments for the author

1. Can the authors comment about several of the other severely affected pathways in Fig. 2, such as Rel, Lat, Lck. These seem to be affected much more so than Igf-1r and IR.

2. In Fig. 3, Chd5-CreER should delete IGF-1 broadly in blood vessels throughout the animal. Why then is vessel-derived IGF-1 not required for forelimb cartilage growth plates or other structures? Is this a timing issue? If not, further discussion is warranted.

3. In Fig 4C,D - how was "directly adjacent to implanted bead" determined for the quantification of proliferation?

4. The title of the manuscript could be changed to "vessel-derived Igf1" from "angiocrine Igf1" to be more specific.

Reviewer 3

Advance summary and potential significance to field

This paper reports that deficient IGF-1 signalling results in hypoplastic growth of Meckel's cartilage.

Comments for the author

Much of this data does not seem entirely novel as aorta conditioned media was shown to elicit these responses in previous publications and IGF mutants have previously been characterized.

© 2020. Published by The Company of Biologists under the terms of the Creative Commons Attribution License (https://creativecommons.org/licenses/by/4.0/).

Initial rationale for focusing on IGF-1 is not convincing. Would Aorta conditioned media without IGF elicit the same response?

Despite the authors stating that "Removal of IGF-1 specifically in chondrocytes (Col2a1-Cre; Igf-1fl/fl) causes only mild skeletal defects in adult mice, suggesting an endocrine/paracrine requirement for IGF-1 in controlling chondrocyte growth during development.', the other likely explanation is that other factors are likely essential for chondrocyte growth. This latter explanation is supported by characterization of the aorta conditioned media and recognized by the authors " This suggests ligands for FGFR1 and EGFR, such as FGFs EGF and TGF-a, may serve as additional angiocrine factors important for craniofacial development".

Authors are equating jaw length with Meckel's length, which is not necessarily accurate. An alizarin red stain would be needed to make that conclusion

---

First revision

Author response to reviewers' comments

Response to Reviewers:

Editor's Note:

1. Provide a detailed description (in Results) or a revised legend to help the reader to navigate through the data presented in Figure 1A-D.
We have provided additional information in both the Results and Figure Legend to clarify the data presented in Figure 1A-D.

2. Inference of "closely mirrored the IGF-1R signalling component activate in ATDc5 cells with recombinant IGF-1"": Comment on the differences in the phosphorylation (timing and level) of p-IGF- IR Y1136, p-S6K T389 and p-Fox03a T32 between treatment of aorta-conditioned medium and recombinant IGF-1 (figure 1E-F)
We now include a comment on the difference in timing and activation of IGF-1R and downstream signalling components between the two different treatment conditions, and discuss possible reasoning for the differences observed (including differing concentrations of IGF-1 and/or presence of modifying proteins such as IGF binding proteins (IGFBPs).

3. Figure 2C and text: Clarify if ps6k-T389 (with reduced phosphorylation) is same as p70S6K (data not shown?), and if phosphorylation of ERK1/2 was significantly reduced. It would be informative to quantify the level of phosphorylation of the factors shown in Fig 2C.
pS6K-T389 and p70S6K (data not shown) are indeed the same protein. We have now modified Figure 1 and Figure 2 to label p-S6K T389 as p-P70S6K T389.
We now present quantitation of Western blots shown in Figure 2C as fold change (KO/WT), which is shown in a panel at the right of Figure 2C. ERK1 showed 0.738 reduction in phosphorylation, whereas ERK2 was not significantly changed (0.918). This has now been amended accordingly.

4. Comment on the phenomenon of lack of effect of IGF-1 signals from the investing mesenchyme (Fig S2) on the growth of the Meckel's cartilage of the endothelial-IGF1 deficient Cdh5-CreERT2;Igf1-f/f embryo
We now include a comment on this in the discussion.
"As well as by the vasculature, *Igf-1* is expressed by the investing mesenchyme in close proximity to Meckel's cartilage (Supp. Fig. 2), which remains highly expressed in *Cdh5-CreERT2; Igf-1*$^{fl/fl}$ embryos. Why this expression does not compensate for lack of IGF-1 in endothelial cells remains to be investigated, but may involve the activity and expression of IGFBPs which can enhance or attenuate the signalling activity of IGFs, and remain to be studied in the context of Meckel's cartilage development."

© 2020. Published by The Company of Biologists under the terms of the Creative Commons Attribution License (https://creativecommons.org/licenses/by/4.0/).

Reviewer 1 Advance Summary and Potential Significance to Field:
Angiocrine regulation of Igf1 might promote bone repair and regeneration.

Reviewer 1 Comments for the Author:
This is a well executed study demonstrating the angiocrine function of Igf1 in bone development worthy of publication.

Reviewer 2 Advance Summary and Potential Significance to Field:
This is a well performed and rigorous study. It clearly demonstrates an interesting role for vessel- derived IGF-1 in promoting growth of Meckel's cartilage and hence the lower jaw. A number of sophisticated techniques are employed. Proteomic arrays identified Igfr-1 signaling and its downstream effectors in Meckel's cartilage, which are well validated from Westerns. Mouse genetics identify that Igfr-1 signaling depends on the presence of sufficient blood vessels, and conditional deletion of Igf-1 in the vasculature clearly reveals a role for vessel-derived Igf-1 in Meckel's growth. Lastly, addition of Igf-1 to Meckel's explants rescues growth in vessel-deficient jaws. I found the paper to be an elegant example of how vessel-derived signals potentiate jaw growth and had only very minor comments.

Reviewer 2 Comments for the Author:
1. Can the authors comment about several of the other severely affected pathways in Fig. 2, such as Rel, Lat, Lck. These seem to be affected much more so than Igf-1r and IR.
We have now included a comment on these severely affected substrates in the Discussion.

2. In Fig. 3, Chd5-CreER should delete IGF-1 broadly in blood vessels throughout the animal. Why then is vessel-derived IGF-1 not required for forelimb cartilage growth plates or other structures? Is this a timing issue? If not, further discussion is warranted.
We have included a comment on this in the Discussion, addressing possible differences in chondrocytes of neural crest versus mesenchymal developmental origin.

3. In Fig 4C,D - how was “directly adjacent to implanted bead” determined for the quantification of proliferation?
We have amended the methods section to define the region of chondrocytes directly adjacent the implanted bead (200□m radius from the bead).

4. The title of the manuscript could be changed to "vessel-derived Igf1" from "angiocrine Igf1" to be more specific.
This is a good suggestion. We have now changed the title accordingly. The title now reads “Vessel- derived angiocrine IGF-1 promotes Meckel’s cartilage proliferation to drive jaw growth during embryogenesis”

Reviewer 3 Advance Summary and Potential Significance to Field:
This paper reports that deficient IGF-1 signalling results in hypoplastic growth of Meckel's cartilage.

Reviewer 3 Comments for the Author:
Much of this data does not seem entirely novel as aorta conditioned media was shown to elicit these responses in previous publications and IGF mutants have previously been characterized.
While we have previously shown aorta-conditioned media induces chondrocyte proliferation, we show for the first time in this manuscript that IGF-1 is present in aorta-conditioned media and responsible for the proliferative effects. We agree that skeletal defects in several IGF-1 mutants have been previously characterised by others, but the specific source of IGF-1 was unknown. The novelty of our data lies in the first description of an endothelial-specific knockout of IGF-1, and it’s associated craniofacial defects. This is the first evidence of an angiocrine role for IGF-1 in development and uncovers new mechanistic insight to how vessels impact craniofacial morphogenesis.

Initial rationale for focusing on IGF-1 is not convincing. Would Aorta conditioned media without IGF elicit the same response?
Our initial rationale was based on the findings from an unbiased screen showing that aorta

© 2020. Published by The Company of Biologists under the terms of the Creative Commons Attribution License (https://creativecommons.org/licenses/by/4.0/).

conditioned media primarily activates the Insulin / IGF signalling pathway in a chondrogenic cell line. We have not explicitly tested that removal of IGF-1 from aorta-conditioned media ameliorates the proliferative effects. While this could be pursued with function blocking antibodies, such an analysis would be redundant in light of conclusive data presented in Figures 2-4 (ie. (1) Proteomics signalling array on primary cartilage tissue, and (2) Conditional removal of IGF-1 from blood vessels).

Despite the authors stating that "Removal of IGF-1 specifically in chondrocytes (Col2a1-Cre; Igf-1fl/fl) causes only mild skeletal defects in adult mice, suggesting an endocrine/paracrine requirement for IGF-1 in controlling chondrocyte growth during development.', the other likely explanation is that other factors are likely essential for chondrocyte growth. This latter explanation is supported by characterization of the aorta conditioned media and recognized by the authors " This suggests ligands for FGFR1 and EGFR, such as FGFs, EGF and TGF-a, may serve as additional angiocrine factors important for craniofacial development".
The lack of a skeletal phenotype in *Col2a1-Cre; Igf-1$^{fl/fl}$* demonstrates that IGF-1 is not required in a cell-autonomous manner by the chondrocytes for cartilage development. IGF-1, however, is indeed essential for skeletal development, as full Igf-1 knockout mice have very severe skeletal defects.

Also, loss of the IGF-1R in chondrocytes (*Col2a1-Cre; IGF-1R$^{fl/fl}$* mice) causes skeletal phenotypes, demonstrating chondrocytes require IGF-1 signalling for their development from another source. Taken together, this previous data, and our current data in this manuscript, support a paracrine role for IGF-1 acting on chondrocytes for their development. We have modified our text in the Discussion to further clarify and emphasise these points.
We agree with the reviewer that many other factors are likely important for cartilage growth, and for this reason discuss this possibility in our manuscript.

Authors are equating jaw length with Meckel's length, which is not necessarily accurate. An alizarin red stain would be needed to make that conclusion
We and others have demonstrated that Meckel's cartilage shape and length is directly proportional to the shape and length of the mandible (jaw) bone, as Meckel's cartilage provides a scaffold for mandible ossification. We agree that Meckel's cartilage and the mandible bone are different structures, and the absolute length of each structure is not equal. However, the length of these structures are always directly proportional to each other. We have modified our manuscript text to emphasise this point. Our figures are labelled as measuring "Meckel's cartilage length" and not "jaw length" accordingly.

Second decision letter

MS ID#: DEVELOP/2020/190488

MS TITLE: Vessel-derived angiocrine IGF-1 promotes Meckel's cartilage proliferation to drive jaw growth during embryogenesis

AUTHORS: Ceilidh Marchant, Peter Anderson, Quenten Schwarz, and Sophie Wiszniak
ARTICLE TYPE: Research Report

I am happy to tell you that your manuscript has been accepted for publication in Development, pending our standard ethics checks.

© 2020. Published by The Company of Biologists under the terms of the Creative Commons Attribution License (https://creativecommons.org/licenses/by/4.0/).
